# *Breynia phongquangensis* (Phyllanthaceae), a new species from Vietnam

**DOI:** 10.3897/phytokeys.275.195982

**Published:** 2026-05-27

**Authors:** Ngoc Han Le, The Bach Tran, Van Hai Do, Duc Binh Tran, Thi Hoan Duong, Thu Ha Bui

**Affiliations:** 1 Institute of Biology, Vietnam Academy of Science and Technology, 18 Hoang Quoc Viet, Nghia Do, Ha Noi, Vietnam Institute of Biology, Vietnam Academy of Science and Technology Ha Noi Vietnam; 2 Graduate University of Science and Technology, Vietnam Academy of Science and Technology, 18 Hoang Quoc Viet, Nghia Do, Ha Noi, Vietnam Graduate University of Science and Technology, Vietnam Academy of Science and Technology Ha Noi Vietnam; 3 Faculty of Biology, Hanoi National University of Education, 136 Xuan Thuy, Cau Giay, Ha Noi, Vietnam Faculty of Biology, Hanoi National University of Education Ha Noi Vietnam

**Keywords:** *
Glochidion
*, Phong Quang, *
Phyllanthus
*, *
Synostemon
*, Tuyen Quang

## Abstract

The new species *Breynia phongquangensis* from Vietnam is described and illustrated. The new species is compared with three similar species in an identification key and a table of detailed morphological characters. *Breynia phongquangensis* differs from the morphologically similar species *B. macrantha*, *B. phuongiana*, and *B. spatulifolia* by a unique combination of characters, including fleshy sepals in the staminate flowers (vs. non-fleshy in the other three species); staminate flowers 4.9–5.8 mm in diameter (vs. ca. 3.3 mm in *B. spatulifolia*); outer sepals of pistillate flowers 3.7–4.6 mm long (vs. 6–7 mm long in *B. macrantha*); and a markedly longer ovary, 3.7–4.9 mm long (vs. < 2.8 mm long in the other three species). It further differs in having fruits ca. 27.0 mm long (vs. ≤ 25.0 mm long in the other species), pistillate flower pedicels 16–53 mm long (vs. 2.5–4.0 mm long in *B. phuongiana* and 0.3–3.0 mm long in *B. spatulifolia*), globose fruits (vs. starfish-shaped in *B. phuongiana*), and fewer lateral veins (6–8 pairs vs. 9–15 pairs in *B. spatulifolia*).

## Introduction

*Breynia* J.R.Forst. & G.Forst. is a genus in the flowering plant family Phyllanthaceae, comprising 74 accepted species distributed from India and China through Southeast Asia to Australia and the western Pacific (POWO 2026). The genera *Breynia*, *Glochidion*, and *Synostemon* were recently found to be nested within *Phyllanthus*, and discussions ensued regarding whether to subsume everything into *Phyllanthus* s.l. (Bouman et al. 2022). Instead of combining all these genera, Bouman et al. (2022) implemented the solution of splitting *Phyllanthus* into strictly monophyletic genera to ensure that the classification is consistent with the latest phylogenetic results. With this new division of the genus *Phyllanthus*, tribe Phyllantheae consists of 18 genera, including *Breynia*, *Phyllanthus*, and 16 others (Bouman et al. 2022).

In Vietnam, 25 species of *Breynia* have been recorded to date (Pham 2003; Nguyen 2000, 2007; Nguyen et al. 2026; POWO 2026). In 2026, during a botanical survey of the Phong Quang Nature Reserve, Tuyen Quang Province, Vietnam, specimens of the genus *Breynia* were collected. After comparison with specimens in the herbaria HN, K, KRIB, P, and VNM and consultation of the relevant literature, these specimens were determined to represent a new species. Here, this new species is described and illustrated as *Breynia phongquangensis* T.H.Bui & N.H.Le.

## Materials and methods

All morphological characters of the new species were observed from living and dried specimens. The material was deposited at the HN Herbarium of the Institute of Biology in Vietnam. The conservation status of the new species was assessed according to the guidelines of the International Union for Conservation of Nature (IUCN 2025). Other specimens of *Breynia* species were studied at the CPNP, HN, KRIB, and VNM herbaria, which preserve many specimens of species distributed in Vietnam (acronyms follow Thiers 2026).

## Taxonomy

### Breynia phongquangensis

Taxon classificationPlantaeSpatangoidaLoveniidae

T.H.Bui & N.H.Le sp. nov.

8407D939-8184-5EF5-A326-8493F462E301

urn:lsid:ipni.org:names:77380714-1

Figs 1, 2

#### Type.

**Vietnam** • Tuyen Quang Province, Minh Tan Commune, Phong Quang Nature Reserve, 22°56'36.90"N, 104°53'26.59"E, alt. 730 m, 22 March 2026, *T.B. Tran, V.H. Do, T.C. Nguyen, T.H. Duong, N.H. Le, N.M. Trinh, A.T. Vu, D.B. Tran*, *VAST-PQ 74* (holotype: HN000083328!; isotypes: HN000083329!, HN000083330!, HN000083331!, HN000083332!, HN000083333!, HN000083334!, HN000083335!, VNM00078331!).

#### Diagnosis.

*Breynia phongquangensis* differs from *B. macrantha*, *B. phuongiana*, and *B. spatulifolia* in having fleshy staminate sepals. It is further distinguished from *B. spatulifolia* by its larger staminate flowers and different number of lateral veins; from *B. macrantha* by the shorter outer sepals of the pistillate flowers; and from *B. phuongiana* by its distinct fruit shape. In addition, *B. phongquangensis* possesses longer ovaries and fruits than all three allied species, as well as longer pistillate flower pedicels than those of *B. phuongiana* and *B. spatulifolia*.

**Figure 1.**
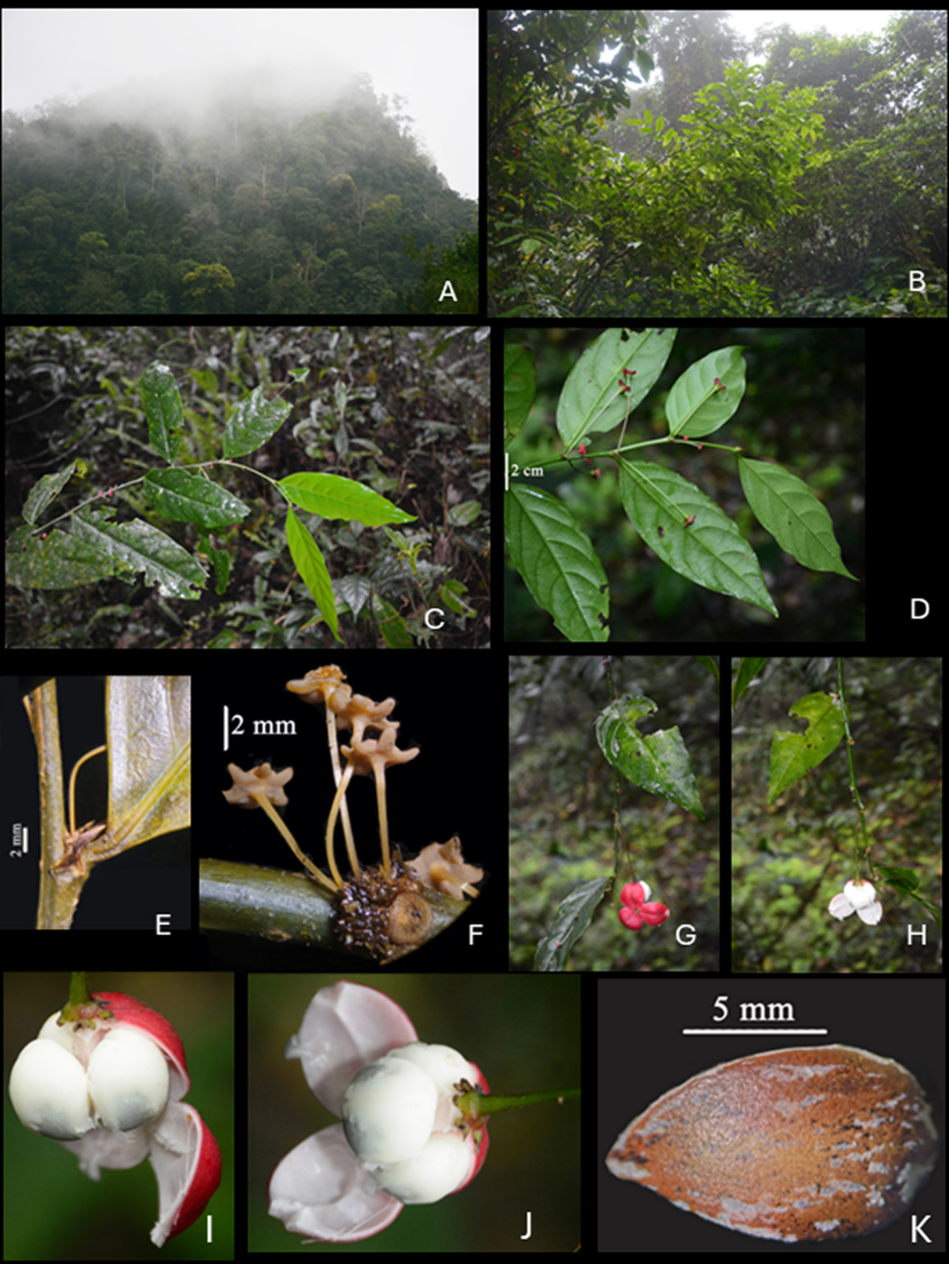
*Breynia phongquangensis* T.H.Bui & N.H.Le. **A**. Habitat; **B**. Habit; **C**. Flowering branch viewed from the upper side of the leaf; **D**. Flowering branch viewed from the lower side of the leaf; **E**. Section of the branch showing stipules; **F**. Inflorescence; **G, H**. Fruiting branch; **I, J**. Fruit; **K**. Seed with white testa removed. Photos by T.B.Tran & A.T.Vu.

#### Description.

***Shrubs*** 4–5 m tall, erect, monoecious, glabrous throughout; branchlets slightly zigzag, angular when young, terete with age, slender, green, internodes 21–37 mm long. ***Stipules*** persistent, triangular with a long acumen, 3.5 × 1.0 mm, greenish-brown. ***Leaves*** simple, distichous, short petiolate; petiole 3–5 mm long; leaf blade ovate, obovate, elliptic to oblong-elliptic, lanceolate, 45–153 × 29–52 mm, glabrous at both surfaces, cuneate, retuse or acuminate at base, acuminate to round at apex, with entire margin; venation pinnate with lateral veins in 6–8 pairs, elevated abaxially, reticulate veins obvious, flattened adaxially, unclear interconnected near the margin. ***Inflorescence*** axillary or cauliflorous, 1–6-flowered, peduncle absent or very short; bracts triangular. ***Flowers*** actinomorphic, pedicellate. ***Staminate flowers***: ***pedicel*** slender, 3.5–8.6 mm long, glabrous; ***calyx*** shallowly disk-shaped, 4.9–5.8 cm in diameter, pink-red mottled with white, 6-lobed, thick 0.4–0.7 mm, fleshy; ***calyx lobe*** apex round to slightly emarginate, 1.0–1.5 × 1.1–1.7 mm; two surfaces mottled with white; ***androphore*** pale yellow, very short, 0.1–0.2 mm long; ***stamens*** three, inserted at lobe apex; anthers 0.36–0.41 mm long, dehiscing by longitudinal slits, extrorse. ***Pistillate flowers*** 0.43–0.66 cm in diameter; ***pedicel*** 16–53 mm long; ***calyx*** pink-red mottled with white, 6-lobed; *sepals* with two types (biseriate), three inner sepals broadly ovate, 3.0–3.2 × 3.0–3.6 mm, three outer sepals broadly obovate, apex emarginate, 3.7–4.6 × 2.5–3.6 mm. ***Ovary*** in transversal section nearly circular, 3.7–4.9 mm in diameter, with three blunt angles, 3-locular; stigmas three, bifid up to 3/4 their length, recurved; ovules two per locule, white. ***Fruit*** globose, 27 mm in diam­eter, when open composed of three free lobes, each lobe has a vertical stripe down the middle, outer fruits pink-red, inner fruits white, calyx persistent. ***Seeds*** 3-sided, each side surface ovate, glabrous, white, 22–23 × 13–14 mm.

**Figure 2.**
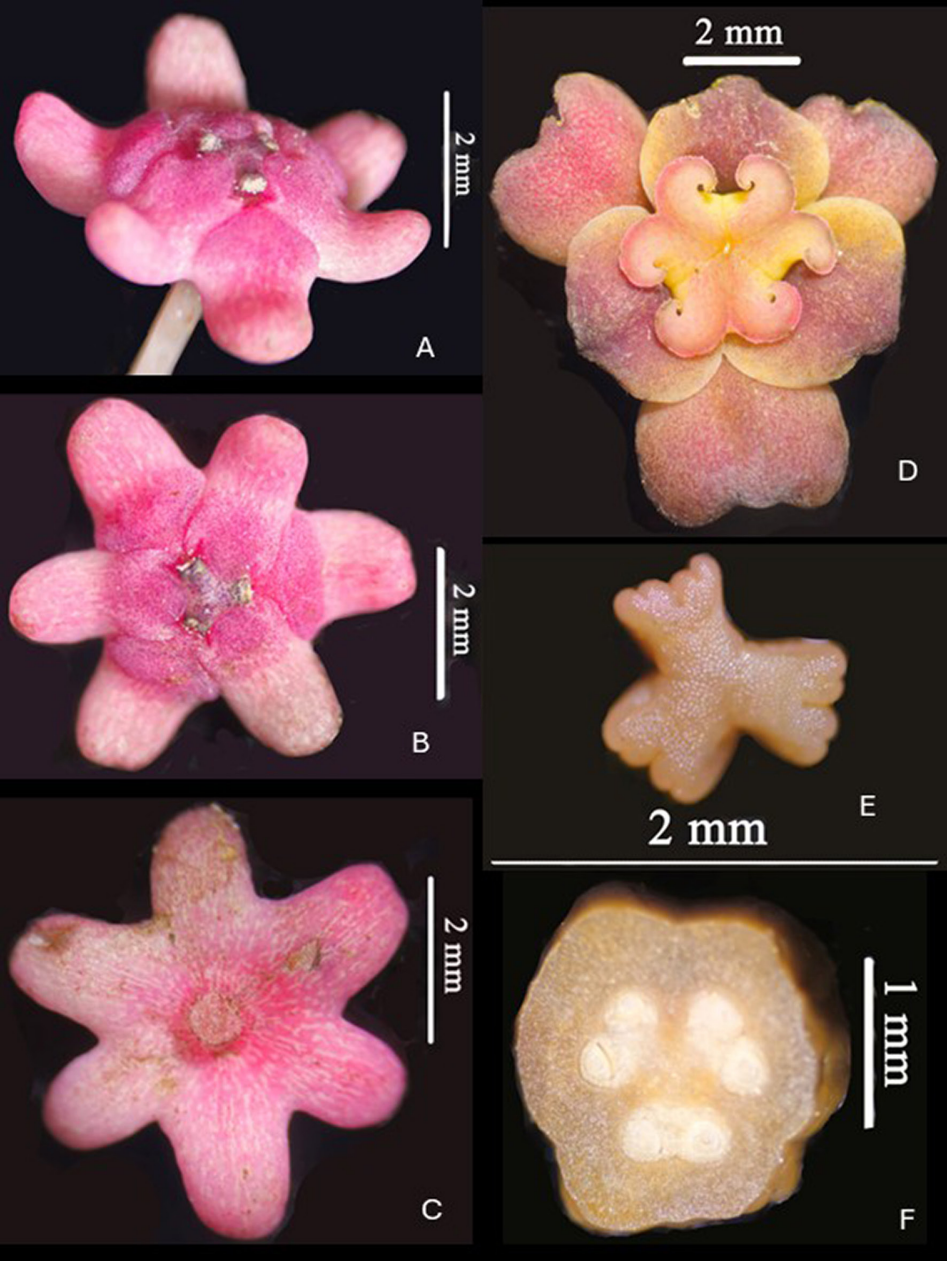
*Breynia phongquangensis* T.H.Bui & N.H.Le. **A**. Staminate flower viewed from the side; **B**. Staminate flower viewed from above; **C**. Staminate flower viewed from below; **D**. Pistillate flower viewed from above; **E**. Stamens; **F**. Cross section of ovary. Photos by T.B.Tran & A.T.Vu.

#### Etymology.

The specific epithet refers to the type locality, Phong Quang Nature Reserve, Tuyen Quang Province, in Vietnam.

#### Distribution and ecology.

*Breynia phongquangensis* is found only in Vietnam, Tuyen Quang Province, Phong Quang Nature Reserve, where it grows in the evergreen broadleaf forests on a limestone mountain in association with *Burretiodendron tonkinense* (A.Chev.) Kosterm., *Claoxylon indicum* (Reinw. ex Blume) Hassk., *Euonymus bockii* Loes. ex Diels, *Fissistigma latifolium* (Dunal) Merr., *Hoya vangviengiensis* Rodda & Simonsson, *Micromelum hirsutum* Oliv., *Miliusa balansae* Finet & Gagnep., and *Vanilla somai* Hayata. Flowering and fruiting specimens were collected in March and April 2026.

#### Conservation status.

*Breynia phongquangensis* is currently known only from its type locality in Phong Quang Nature Reserve, Tuyen Quang Province, and its occurrence in other areas has not yet been evaluated. During field observations, no immediate threats to the species were observed within the nature reserve. The locality also harbors several threatened species, including *Burretiodendron tonkinense*, assessed as Near Threatened (NT) (IUCN 2025) and Endangered (EN) (Tran et al. 2024); and *Vanilla somai*, assessed as Endangered (EN) (IUCN 2025), highlighting the conservation importance of this area. However, *B. phongquangensis* inhabits rugged rocky montane habitats that are difficult to access, even for botanical surveys. Consequently, the population size and number of mature individuals remain unknown. Therefore, the species is here assessed as Data Deficient (DD) according to the IUCN Red List Categories and Criteria (IUCN 2025).

## Discussion

Four species were chosen for comparison because they possess distinctive characteristics that set them apart from the others: first, flowers ranging from pink to red, closely resembling the pink, red, or purple found in the flowers of all four species; and second, pistillate flower sepals of two types (biseriate), the first type with three inner sepals and the second type with three outer sepals.

Diagnostic characters separating the allied species are listed in Table 1. Based on the keys and species descriptions of *Breynia* provided in previous studies (Beille 1927; Chayamarit et al. 2005; van Welzen 2007; Li et al. 2008; Nguyen et al. 2026), the new species is distinguished by the following combination of characters: staminate flower calyx fleshy; staminate flowers 4.9–5.8 mm in diameter; outer sepals of pistillate flowers 3.7–4.6 mm long; ovary 3.7–4.9 mm long; fruits 27.0 mm long and globose; pedicels of pistillate flowers 16–53 mm long; and lateral veins 6–8 pairs.

**Table 1.** Morphological comparisons of *Breynia phongquangensis* with similar species.

Characters	*B. phongquangensis*	*B. macrantha* (Pham 2003; van Welzen 2007; Li et al. 2008)	*B. phuongiana* (Nguyen et al. 2026)	*B. spatulifolia* (Pham 2003; van Welzen 2007; Li et al. 2008)
Leaf shape and size (mm)	ovate, obovate, elliptic, oblong-elliptic, lanceolate, 45–153 × 32–52	ovate, ovate-oblong, ovate-elliptic, elliptic-lanceolate, 40–200 × 35–80	elliptic, oblong-elliptic, 170–175 × 57–61	oblong, 76–130 × 22–38
Lateral veins (pairs)	6–8	5–10	7–12	9–15
Petiole length (mm)	3.0–5.0	3.0	3.0–4.0	2.0–4.5
Stipule shape, size	triangular with a long acumen, 3.5 × 1.0 mm	triangular, subulate-lanceolate, 3.0–8.5 × 2–2.5 mm long	ovate with a long tail, 4.0–6.0 × 2.5–3.0 mm	triangular, 5.0–6.0 × ca. 4.0 mm
Inflorescences position	axillary or cauliflorous	axillary	cauliflorous	cauliflorous
Staminate flowers (pedicel length, flower diameter, fleshy/not fleshy sepals)	pedicel 3.5–8.6 mm long, flower 4.9–5.8 mm in diameter, sepals fleshy	pedicels 2.0–6.5 mm long, flower 3.5–4.5 mm in diameter, sepals not fleshy	pedicel 2.0–5.0 mm long, flower 7.0–7.5 mm in diameter, sepals not fleshy	pedicel 3.0–5.0 mm long, flower ca. 3.3 mm in diameter, sepals not fleshy
Staminate flower color	pink-red mottled with white	yellow-red, red stripes	red mottled with white	red, purple
Pistillate flowers (pedicel length, flower diameter)	pedicel 16.0–53.0 mm long, flower 4.3–6.6 mm in diameter	pedicels 20.0–63.0 mm long, flower 6.0–14.0 mm in diameter	pedicel 2.5–4.0 mm long, flower 8.0–8.5 mm in diameter	pedicel 0.3–3.0 mm long, flower 4.0–4.8 mm in diameter
Pistillate flowers (sepal number, shape, size)	sepals 6, biseriate, three inner sepals broadly ovate, 3.0–3.2 × 3.0–3.6 mm; three outer sepals broadly obovate, 3.7–4.6 × 2.5–3.6 mm	sepals six, biseriate, inner sepals shorter, obovate, ca. 5 mm long; outer sepals obovate, subspatulate, ovate-elliptic, 6–7 × 3–4 mm	sepals 6, biseriate, three inner sepals broadly ovate, 1.18–1.73 × 2.23–2.41 mm; three outer sepals broadly, 1.59–2.31 × 2.29–2.45 mm	sepals 6, biseriate, subequal, obovate, 2–3 × ca. 1.5 mm
Pistillate flower color	pink-red mottled with white	red, yellow-green, with purple stripes	red mottled with white	red, purple
Pistillate pistil (shape, size)	ovary in transversal section nearly circular, 3.7–4.9 mm in diameter	ovary depressed globose, ca. 2.0 × 2.7 mm	ovary in transversal section triangular with three blunt angles, ca. 2 × 2 mm	ovary bell-shaped, ca. 0.8 × 0.7–0.8 mm
Fruit (shape, size)	globose, 27.0 mm in diameter	depressed globose, 10.0–15.0 × 15.0–25.0 mm	starfish-shaped, 23.0 mm in diameter	globose, ca. 7.0 × 5.5 mm

### Identification key to a group of four similar species of *Breynia* including *B. phongquangensis*

**Table:** 

1	Staminate flower sepals fleshy	***B. phongquangensis***
–	Staminate flower sepals not fleshy	**2**
2	Fruit starfish-shaped	***B. phuongiana***
–	Fruit depressed globose to globose	**3**
3	Pistillate pedicels 20.0–63.0 mm long, flower 6.0–14.0 mm in diameter	***B. macrantha***
–	Pistillate pedicel 0.3–3.0 mm long, flower 4.0–4.8 mm in diameter	***B. spatulifolia***

**Additional specimens examined (paratypes). Vietnam** • Tuyen Quang Province, Minh Tan Commune, Phong Quang Nature Reserve, 13 April 2025, *H.Q. Bui, T.T.H. Nguyen, T.H. Duong, N.H. Le, T.T. Nguyen, D.B. Tran, VAST-PQ 09* (HN).

## Supplementary Material

XML Treatment for Breynia phongquangensis

## Additional information

### Artificial Intelligence (AI) use

The authors accept full responsibility for the content of the manuscript, including the disclosure of any use of AI.

No AI tools were used in the preparation of this manuscript.

### Author ORCIDs

N.H. Le https://orcid.org/0000-0003-2657-5091

T.B. Tran https://orcid.org/0000-0003-3798-5969

V.H. Do https://orcid.org/0000-0001-7841-0585

D.B. Tran https://orcid.org/0000-0001-6658-0739

T.H. Duong https://orcid.org/0000-0002-4298-5720

T.H. Bui https://orcid.org/0000-0001-6971-0105

### Data availability

All of the data that support the findings of this study are available in the main text.
